# Behavioral Weight Loss Programs for Cancer Survivors Throughout Maryland: Protocol for a Pragmatic Trial and Participant Characteristics

**DOI:** 10.2196/54126

**Published:** 2024-06-12

**Authors:** Gerald J Jerome, Lawrence J Appel, Linda Bunyard, Arlene T Dalcin, Nowella Durkin, Jeanne B Charleston, Norma F Kanarek, Michael A Carducci, Nae-Yuh Wang, Hsin-Chieh Yeh

**Affiliations:** 1 Department of Kinesiology Towson University Towson, MD United States; 2 Department of Medicine Johns Hopkins University Baltimore, MD United States; 3 Department of Epidemiology Johns Hopkins University Baltimore, MD United States; 4 Department of Environmental Health and Engineering Johns Hopkins University Baltimore, MD United States; 5 Sidney Kimmel Comprehensive Cancer Center Johns Hopkins University Baltimore, MD United States; 6 Department of Biostatistics Johns Hopkins University Baltimore, MD United States

**Keywords:** cancer, obesity, weight loss, pragmatic trial, oncology, weight, overweight, obese, USA, United States, survivor, survivors, remote, self-directed, self-guided, coach, coaching, mHealth, mobile health, app, apps, application, applications, EHR, health record, health records, mobile phone

## Abstract

**Background:**

Clinical trials examining lifestyle interventions for weight loss in cancer survivors have been demonstrated to be safe, feasible, and effective. However, scalable weight loss programs are needed to support their widespread implementation. The ASPIRE trial was designed to evaluate real-world, lifestyle-based, weight loss programs for cancer survivors throughout Maryland.

**Objective:**

The objectives of this protocol paper are to describe the design of a nonrandomized pragmatic trial, study recruitment, and baseline characteristics of participants.

**Methods:**

Participants were aged ≥18 years, residing in Maryland, with a BMI ≥25 kg/m^2^, who reported a diagnosis of a malignant solid tumor, completed curative treatment, and had no ongoing or planned cancer treatment. Enrollment criteria were minimized to increase generalizability. The primary recruitment source was the Johns Hopkins Health System electronic health records (EHRs). Participants selected 1 of 3 remotely delivered weight loss programs: self-directed, app-supported, or coach-supported program.

**Results:**

Participants were recruited across all 5 geographic regions of Maryland. Targeted invitations using EHRs accounted for 287 (84.4%) of the 340 participants enrolled. Of the 5644 patients invited through EHR, 5.1% (287/5644) enrolled. Participants had a mean age of 60.7 (SD 10.8) years, 74.7% (254/340) were female, 55.9% (190/340) identified as non-Hispanic Black, 58.5% (199/340) had a bachelor’s degree, and the average BMI was 34.1 kg/m^2^ (SD 5.9 kg/m^2^). The most common types of cancers were breast (168/340, 49.4%), prostate (72/340, 21.2%), and thyroid (39/340, 8.5%). The self-directed weight loss program (n=91) included 25 participants who agreed to provide weights through a study scale; the app-supported program (n=142) included 108 individuals who agreed to provide their weight measurements; and the coach-supported weight loss program included 107 participants. We anticipate final analysis will take place in the fall of 2024.

**Conclusions:**

Using EHR-based recruitment efforts, this study took a pragmatic approach to reach and enroll cancer survivors into remotely delivered weight loss programs.

**Trial Registration:**

ClinicalTrials.gov NCT04534309; https://clinicaltrials.gov/study/NCT04534309

**International Registered Report Identifier (IRRID):**

DERR1-10.2196/54126

## Introduction

Obesity is a major public health concern that adversely affects cardiometabolic health [1-3]. Less well appreciated is that obesity increases the risks of some cancers [4]. In addition, obesity is associated with cancer recurrence. A meta-analysis showed obesity was associated with increased risks of recurrence in breast, colorectal, prostate, and esophageal cancers [5]. Furthermore, certain cancer treatments lead to weight gain [6]. Hence, for many cancer survivors, obesity poses a dual risk of decreased cardiometabolic health and increased cancer recurrence.

In the United States, there were an estimated 18 million cancer survivors in 2022 [7], and this number is expected to increase to 22.5 million by 2032 due to advancements in treatment and early detection [8]. It is estimated that 32.5% of cancer survivors are overweight or obese [7]. Scalable weight loss interventions are needed to help cancer survivors reduce their cardiometabolic risk, lower their risk of cancer recurrence, and improve their long-term health profile.

The International Agency for Research on Cancer and the American Cancer Society recommend a lifestyle approach that includes diet and physical activity to avoid weight gain and maintain a healthy body weight, which can reduce the risk of cancer recurrence [9,10]. Over the past decade, trials examining lifestyle interventions for weight loss in cancer survivors have been demonstrated to be safe, feasible, and effective [11]. However, there is a need to implement effective programs at scale and to enroll a diverse population [11]. With these long-term goals in mind, we intended to implement scalable approaches to providing lifestyle-based weight loss programs for cancer survivors throughout Maryland, including its rural communities.

Many trials, including several by our team, have demonstrated the effectiveness and feasibility of remotely delivered weight loss programs. For example, the POWER trial at Johns Hopkins, a comparative effectiveness trial, was one of the first large-scale, long-term weight loss trials to demonstrate that a remote weight loss program could be as effective as an in-person weight loss program [12]. Specifically, individuals who received in-person support and remote support achieved a 5.2% and 5% decrease in weight at 24 months, respectively. Subsequently, we tailored the remote weight loss program for cancer survivors and found it to be effective [13,14]. This study takes an important next step in translating clinical trial results into more scalable programs that could be used to disseminate and implement these programs.

The ASPIRE trial was designed to evaluate real-world, lifestyle-based, weight loss programs for cancer survivors throughout Maryland. In this protocol paper, we describe the design of this nonrandomized pragmatic trial, recruitment and enrollment, and baseline characteristics of participants.

## Methods

### Study Overview

The objective of the ASPIRE study was to design, implement, and evaluate lifestyle-based weight loss programs to support cancer survivors with overweight or obesity throughout Maryland. This protocol paper describes the design of the ASPIRE study, the feasibility of recruiting a diverse sample using electronic health records (EHRs), and the sample characteristics, to demonstrate the diversity of this study’s population. The ASPIRE study offered three programs: (1) self-directed weight loss (SDWL), (2) app-supported weight loss (ASWL), and (3) coach-supported weight loss (CSWL). Participants self-selected a program. Eligibility and interest were determined via self-report on surveys and screening phone calls. Those who opted for weight tracking were mailed a scale with the cellular capability to transmit their weights. The primary outcome is the 6-month weight change in the CSWL program. Secondary outcomes include examining weight change among those with scales in the ASWL and SDWL programs.

We designed this study to maximize generalizability through several approaches. First, this study was not restricted to patients with a specific solid tumor. Rather, we designed the interventions to be suitable for cancer survivors with overweight or obesity who can benefit from behavioral weight loss. Second, we minimized the inclusion and exclusion criteria to reduce barriers to participating in a clinical trial. Third, the entire study was conducted remotely, which allowed for recruitment throughout the state, including both urban and rural areas; it also allowed for study assessments during COVID-19 restrictions. Our previous weight loss trials generally required in-person weight assessments, thereby limiting recruitment efforts to the greater Baltimore area [12-14]. By leveraging technology such as email, web-based surveys, study scales that use cellular technology for data transmission, and a commercially available weight loss app, the current study design could be implemented across the state and included weight loss programs that had low marginal costs and were readily scalable.

### Ethical Considerations

The Institutional Review Board at Johns Hopkins School of Medicine reviewed and approved the protocol (IRB00229163). All participants provided informed consent. This study was conducted per the tenets of the Declaration of Helsinki. The background, procedures, and aims of this study were provided to prospective participants before the consent process. Confidentiality was protected by assigning participants unique study identification. To acknowledge the time and effort they dedicated to participating in this study, participants who tracked their weight with study scales were offered a US $25 gift card for completing each follow-up data collection (months 3, 6, and 12) that consisted of a set of study weights and web-based surveys.

### Study Population

This study included cancer survivors aged 18 years or older, residing in Maryland, with a BMI of ≥25 kg/m^2^ (≥23 kg/m^2^ for Asians), who self-reported a previous diagnosis of malignant solid tumor, had completed curative intent treatment, and had no ongoing or planned active treatment (surgery, radiation therapy, or chemotherapy other than chemoprophylaxis). Major inclusion criteria also included: completion of all required surgical, chemotherapy, or radiation curative intent therapy at least 3 months before enrollment; anticipated treatment-free life span of 12 months or longer (study physician assessment based on self-reported cancer type and stage at diagnosis, treatments, and comorbidities); weight ≤400 lb (limitation due to maximum weight obtainable from study scales); and had an email address for regular personal use. Chemoprophylaxis with tamoxifen or aromatase inhibitors for breast cancer in women was permitted, as was anti-luteinizing hormone-releasing therapy for prostate cancer in men. Major exclusion criteria included receiving any chemotherapy (unless antihormonal therapy) or radiation 3 months or less before the proposed program date, and women who were breastfeeding, pregnant, or planning pregnancy within the next year. These were the inclusion or exclusion criteria to qualify for the SDWL program.

### Additional Criteria for the ASWL Program

To qualify for the ASWL program, patients must also meet the following criteria: have a smartphone for personal use; sufficient data plan or internet to support daily use of a weight loss app; and a willingness to track weight, diet, and physical activity through a commercial app (Lose it!).

### Additional Criteria for the CSWL Program

In addition to the above criteria to qualify for the CSWL program, participants must also have a willingness to lose weight by changing diet and physical activity habits; a willingness to complete coaching calls (12 weekly calls and 3 monthly calls); and a sufficient call plan to support coaching calls. All participants in the CSWL program and a self-selected subgroup of SDWL and ASWL participants who elected to receive a study scale were required to meet an additional set of study criteria. The additional eligibility criteria for participants using a study scale included the willingness to record or transmit quarterly weights for 12 months. Exclusion criteria included self-identification of uncontrolled concurrent medical condition (eg, chronic kidney disease receiving dialysis) likely to limit compliance with the program as determined by investigators, current involvement in another organized weight loss program, current use of steroids or other medication known to affect body weight, bariatric surgery scheduled within the next 6 months, and plans to move outside the continental United States in the next 12 months.

### Recruitment

Recruitment strategies and materials were aligned with previous trials that ensured recruitment efforts were appropriate to engage a diverse target population related to biological sex, race, and ethnicity [12-14]. Strategies included flyer distribution to in-state cancer groups, invitations to cancer survivors enrolled in previous studies, prior study participants, and email invitations through MyChart, the Johns Hopkins Medicine EHR patient portal. The use of EHR allowed us to identify potential study participants based on zip code, BMI, race-ethnicity, and previous cancer diagnosis. Specifically, we used EHR to target recruitment in Maryland’s 5 geographic regions: Western Region, Capital Region, Central Region, Southern Region, and Eastern Shore Region [15]. The Western, Southern, and Eastern Shore regions were comprised of rural counties [16]. Using the EHR-based recruitment, we sent email invitations through the MyChart portal to patients in all 5 regions. The MyChart invitation referred interested individuals to a study-specific website that provided participants with general study information, assessed basic eligibility, and served as an entry point for participants to express interest in this study.

### Self-Selection of Weight Loss Program

During screening, participants would indicate which program they were interested in joining. The 3 weight loss programs offered different types of support, required different commitments from the participants, and had different screening criteria. Study staff guided participants toward the program that was best aligned with the participants’ interests and eligibility. For example, those interested in but not eligible for the ASWL program (eg, they did not want to friend study staff on the app) were enrolled in the SDWL program. In this manner, this study provided program options to meet the varying needs and interests of cancer survivors throughout Maryland. Based on study resources, we set the enrollment limit for the CSWL program at approximately 100 participants. Once we reached this target, we deemed enrollment to be complete. The SDWL and the ASWL programs were readily scalable and had no enrollment limits during this study’s enrollment period.

### Weight Loss Programs—Theoretical Framework

All 3 weight loss programs used lifestyle approaches based on social cognitive theory and incorporated behavioral self-management approaches designed to help participants with safe and effective weight loss. These programs were based on previously successful coach-supported weight loss programs [12-14]. The programs had a social cognitive theoretical framework for teaching participants strategies to increase their self-efficacy in making behavior changes [17,18]. This included setting realistic weight loss goals and establishing early success with both process goals (changing behaviors) and outcome goals (losing weight). Specific daily strategies that were encouraged among the participants included self-monitoring, goal-setting, and problem-solving. Problem-solving recommendations took a solution-focused approach [19,20]. The programs were designed to build the participant’s skills and confidence with weight reduction strategies including self-weighing, caloric restrictions, increased physical activity, and self-monitoring. Motivational interviewing was the style of communication used to interact with the participants in the CSWL program [21]. This patient-centered approach was also integrated into the written materials for all 3 programs. In addition, the programs were based on previous programs that ensured materials were appropriate to engage a diverse target population related to biological sex, race, and ethnicity [12-14]. Weight loss recommendations in these programs correspond with weight management guidelines for cancer prevention and cancer survivors [9,22].

### Weight Loss Programs—Descriptions

Participants in the SDWL program received written weight loss group materials by email and no further weight loss program-related contact. These materials included recommendations for a weight loss goal of 5% in 6 months (see Table 1). The materials also included weight loss strategies of caloric restriction, following a Dietary Approaches to Stop Hypertension–aligned diet [23], self-monitoring weight at least weekly, and suggestions to gradually increase to at least 150 minutes per week of moderate-intensity aerobic exercise. All 3 weight loss programs used the same fundamental weight loss materials. Since smoking is a major risk factor for cancer [24], all participants who identified as current smokers received information on the free Maryland Tobacco Quitline [25]. This support was congruent with this study’s overall objective of reducing cardiometabolic risk and risk of cancer reoccurrence.

**Table 1:** Weight loss program summaries.

Program component			Self-directed		App-supported	Coach-supported
**Behavioral weight loss information**						
	Information on general behavioral weight loss strategies for cancer survivors	✓		✓		✓
**Weight goal**						
	5% weight loss in 6 months	✓		✓		✓
**Exercise goal**						
	Build up to 150 minutes per week of aerobic physical activity	✓		✓		✓
**Calorie recommendations**						
	1200 kcal/day if ≤170 lb; 1500 kcal/day if >170 lb and <220 lb; 1800 kcal/day if >220 lb and <270 lb; 2200 kcal/day if >270 lb	✓		✓		✓
**Diet recommendation**						
	Dietary Approaches to Stop Hypertension–like diet	✓		✓		✓
**Weight tracking**						
	Tracking weight at least weekly	✓		✓		✓
**Smoking cessation**						
	Referral to *Maryland Tobacco Quitline* for current smokers	✓		✓		✓
**Weekly support emails**						
	Weekly support emails reinforcing study strategies	None		✓		✓
**Expected app use**						
	Insider’s guide to using the app including tracking weight, physical activity, food, and drink	None		✓		✓
**Study scale**			Optional		Optional	Provided
**Additional weight loss information**						
	Monthly weight loss strategy modules, months 4-6	None		None		✓
**Coaching calls**						
	Weekly calls for months 1-3 (total 12 calls expected); monthly calls for months 4-6 (total 3 calls expected)	None		None		✓

The ASWL program received additional suggestions tailored to the use of a specific free commercially available app, Lose it! (FitNow, Inc). Using a commercially available app supports the participants in the continued use of the app at the end of this study. The ASWL program received directions for signing up for the app and for verifying their app account by friending this study’s profile on the app. They received recommendations on which app features should be used to stay consistent with the evidence-based weight loss recommendation of this study. They also received 25 weekly emails reinforcing the suggested behavioral weight loss strategies.

The CSWL program received the same program features as the ASWL program plus 3 additional monthly weight loss program modules: taking the exercise challenge, eating away from home, and mindful eating. The CSWL program was offered telephonic coaching calls weekly for the first 3 months and monthly for months 4 to 6. Participants in the CSWL program were asked to use this study’s scales for their self-monitoring throughout this study. The coaches had access to this scale data, as well as detailed food tracking data, providing increased accountability.

### Measures

All participants self-reported their type of cancer, basic demographic information, height, and weight. Participants could report more than one type of cancer. Among those receiving study scales, the scales served two purposes: (1) to assess weight as an outcome measure and (2) for regular self-weighing during the weight loss program. The scales (BodyTrace) transmitted weight data to the researchers using cellular technology, did not require the use of the participant’s internet or a mobile phone data plan, and had been used in previous weight loss trials [24,26].

### Power and Sample Size

Given the available coaching resources, we set the enrollment target of the CSWL program at 100 participants. By assuming the correlation between the baseline and the 6-month weight measurements as 0.9, and the SD of baseline weight as 17 kg (conservative assumption based on a previous trial in cancer survivors [14]), we estimated that with 80% power, we could detect a mean weight change of 2.15 kg over a 6-month follow-up within each program using a 2-sided test with type I error of 0.05.

### Data Management

A study database manager maintained this study’s database and merged data collected from the wireless scales with the REDCap (Research Electronic Data Capture; Vanderbilt University) database. Using REDCap, study staff entered data, reviewed participant study status, and regularly monitored data for completeness and accuracy. For example, before enrollment, staff checked the database to ensure that all screening activities had occurred, that the participant met all eligibility criteria, and that all required baseline data had been collected.

### Analytic Plan

We defined the enrollment rate of EHR-based study invitations as the percentage of invitations that resulted in participant enrollment. We described the distribution of baseline characteristics of the participants by the weight loss programs using means and SDs for the continuous variables, and percentages and frequencies for the categorical variables. The primary outcome is weight change from baseline to 6 months by program. Additional analysis will use mixed models including weights from 3, 6, and 12 months of data collection. We anticipate final analysis will take place in the fall of 2024.

## Results

**Figure 1:**
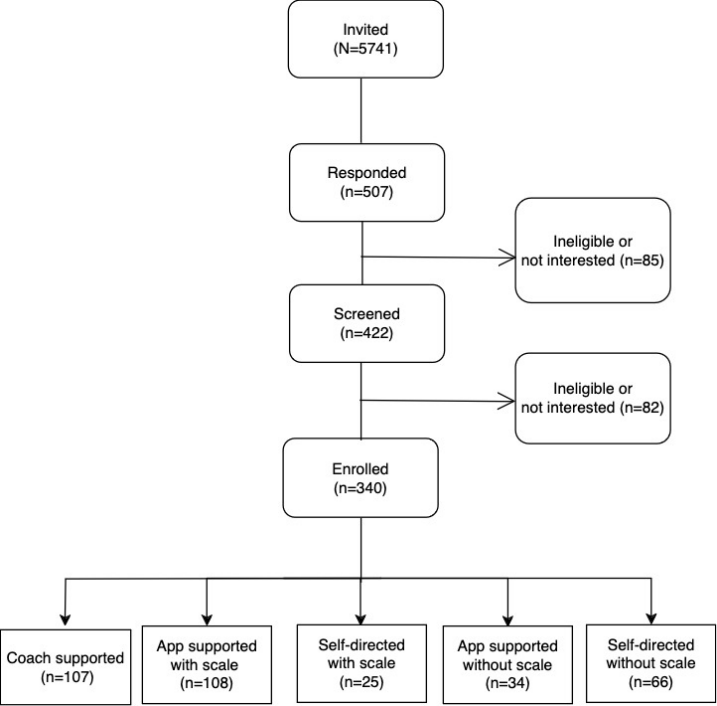
Flow of study participants.

Figure 1 shows the flow of recruitment and enrollment. This study screened 422 individuals between September 2020 and December 2021, of whom 378 were eligible, and 340 enrolled. Targeted invitations using EHR accounted for 84.4% (287/340) of the participants. Of the 5644 invited through the EHR portal, 287 (5.1%) enrolled. Among the 97 previous study participants who were invited, 16 (16.5%) enrolled. We did not have a known denominator from our other recruitment channels and were not able to report on yield for the remaining 37 participants.

As shown in Table 2, the reach ranged from 6.4% (87/1363) in the Capital Region to 4.1% (26/631) in the Western Region. Participants had an average age of 60.7 (SD 10.8) years, were predominantly female (254/340, 74.7%), had an average BMI of 34.1 kg/m^2^ (SD 5.9 kg/m^2^) based on self-reported height and weight, and over half (199/340, 58.5%) had a bachelor’s degree (Table 3). Participants could report more than one type of cancer and the most frequently reported types of cancer were breast cancer (168/340, 49.4%), prostate cancer (72/340, 21.2%), and thyroid cancer (29/340, 8.5%). In terms of race-ethnicity, 55.9% (190/340) of participants were identified as non-Hispanic Black adults and 38.8% (132/340) were identified as non-Hispanic White adults.

**Table 2:** Reach of electronic health record recruitment in Maryland by region^a^.

		Recruitment invitations sent, n	Participants enrolled, n (%)
Total		5644	287 (5.1)
**Region**			
	Capital	1363	87 (6.4)
	Central	1806	95 (5.3)
	Eastern Shore	1027	47 (4.6)
	Southern	739	32 (4.3)
	Western	631	26 (4.1)

^a^Study invitations were sent to those aged 18 years and older, with BMI ≥25 kg/m^2^, a record of a malignant tumor on file, and living within the designated target areas.

**Table 3:** Participant characteristics at baseline.

Characteristics		Total (N=340)	Received a study scale			No study scale	
			Coach supported (n=107)	App-supported (n=108)	Self-directed (n=25)	App-supported (n=34)	Self-directed (n=66)
Age (years), mean (SD)		60.7 (10.8)	59.2 (10.0)	60.7 (10.2)	68.2 (6.4)	56.5 (10.7)	62.5 (12.5)
Female, n (%)		254 (74.7)	87 (81.3)	77 (71.3)	14 (56)	27 (79.4)	49 (74.2)
BMI (kg/m^2^), mean (SD)^a^		34.1 (5.9)	35.1 (6.1)	33.6 (5.2)	31.6 (3.5)	34.5 (6.9)	33.9 (6.3)
**Race or ethnicity, n (%)**							
	Non-Hispanic Black or Latina	190 (55.9)	57 (53.3)	56 (51.9)	13 (52)	25 (73.5)	39 (59.1)
	Hispanic or Latina, not Black	6 (1.8)	1 (0.9)	4 (3.7)	0 (0)	0 (0)	1 (1.6)
	Non-Hispanic or Latina White	132 (38.8)	46 (43)	43 (39.8)	11 (44)	9 (26.5)	23 (34.9)
	Other or unknown	12 (3.5)	3 (2.8)	5 (4.6)	1 (4)	0 (0)	3 (4.5)
**Education, n (%)**							
	Up to some college	99 (29.1)	33 (30.8)	38 (35.2)	3 (12)	13 (38.2)	12 (18.2)
	Bachelor’s degree or higher	199 (58.5)	70 (65.4)	70 (64.8)	22 (88)	17 (50)	20 (30.3)
	Missing or did not answer	42 (12.4)	4 (3.7)	0 (0)	0 (0)	4 (11.8)	34 (51.5)
**Type of cancer^b^, n (%)**							
	Breast	168 (49.4)	63 (58.9)	47 (43.5)	11 (44)	17 (50)	30 (45.5)
	Prostate	72 (21.2)	19 (17.8)	25 (23.2)	10 (40)	6 (17.7)	12 (18.2)
	Thyroid	29 (8.5)	9 (8.4)	11 (10.2)	1 (4)	3 (8.8)	5 (7.6)
	Current smoker	14 (4.1)	5 (4.7)	4 (3.7)	1 (4)	1 (2.9)	3 (4.6)
**Region, n (%)**							
	Capital	94 (27.6)	26 (24.3)	32 (29.6)	9 (36)	9 (26.5)	18 (27.3)
	Central	129 (37.9)	45 (42.1)	36 (33.3)	8 (32)	16 (47.1)	24 (36.4)
	Eastern Shore	55 (16.2)	17 (15.9)	15 (13.9)	4 (16)	4 (11.8)	15 (22.7)
	Southern	34 (10)	7 (6.5)	17 (15.7)	3 (12)	2 (5.9)	5 (7.6)
	Western	28 (8.2)	12 (11.2)	8 (7.4)	1 (4)	3 (8.8)	4 (6.1)

^a^BMI based on self-reported height and weight from prescreening.

^b^Categories are not mutually exclusive.

A direct comparison of the persons enrolled through MyChart to the 5644 who received invitations is not possible, because the list of 5644 was not retained by the data warehouse. However, to overcome this limitation, a repeat search using the same enrollment criteria was carried out, resulting in a contemporary comparison group of 8024 patients who would have met the criteria for potential enrollment; this group was used as an approximate comparison group. Our study participants were younger (mean age 60.7, SD 10.8, vs 70.0, SD 12.9 years, in EHR sample), more likely to be women (74.7% vs 56.3% female in EHR), less likely to be non-Hispanic White (38.8% vs 66% in EHR), had higher BMI (mean BMI 34.1, SD 5.9, vs 31.3, SD 5.4 kg/m^2^, in EHR), and had a lower prevalence of current smokers (4.1% vs 6.7% in EHR).

Among those who enrolled in this study (N=340), 107 individuals selected the CSWL program, 142 selected the ASWL program (of whom 108 were willing to use a study scale to track weight), and 91 selected the SDWL program (of whom 25 were willing to use a study scale). There were differences in participants’ characteristics among programs: participants in the ASWL-nonscale program had an average age of 56.5 (SD 10.7) years and participants in the SDWL-scale program had an average age of 68.2 (SD 6.4) years. The CSWL program had the highest proportion of females (87/107, 81.3%) and the highest average BMI mean, 35.1 kg/m^2^ (SD 6.1 kg/m^2^), while the SDWL with scale had the lowest proportion of females (14/25, 56%) and lowest average BMI mean 31.6 kg/m^2^ (SD 3.5 kg/m^2^). The ASWL-no scale program had the highest proportion of non-Hispanic Black participants (25/34, 73.5%). There were no apparent differences in region by weight loss program. The sample sizes in some programs were modest; hence group comparisons should be interpreted with caution.

## Discussion

In this pragmatic trial, participants self-selected which program they were interested in joining. The 3 weight loss programs offered varying types of support and required different commitments from the participants. Participants who selected the CSWL program tended to be female and with an average BMI of 35.1 (SD 6.1) kg/m^2^. This study demonstrated the ability to recruit a racially diverse sample of cancer survivors who were overweight and with obesity, primarily through an EHR. Among the participants, 56.9% identified as non-Hispanic Black and 38.8% identified as non-Hispanic White. These findings correspond with this study’s objective to recruit a diverse sample and reflect the racial diversity of Maryland where 32% of the population identify as non-Hispanic Black and 48% identify as non-Hispanic White [27].

Recruitment for this pragmatic trial was consistent with our prior success in recruiting racially diverse samples for weight loss studies. Our prior studies have included in-person requirements for study enrollment in urban areas such as Baltimore City, Baltimore County, and Howard County (Central Region) [12-14]. The current study used an all-remote study design and had similar success recruiting in these urban areas. The all-remote design also enabled us to recruit cancer survivors from rural counties in the Western, Southern, and Eastern Shore regions, thereby addressing the need for weight loss programs to support cancer survivors in rural areas [11].

Using EHR-based invitations we were able to target geographic regions throughout Maryland and adjust our recruitment based on program capacity. Although other, more traditional recruitment methods were also used (eg, providing flyers to cancer support groups), EHR-based recruitment accounted for 84% of the participants. In a previous weight loss study for cancer survivors, approximately 54,000 brochures were mailed to targeted zip codes, resulting in 65 enrolled participants or a 0.1% yield [28]. Using Facebook advertisements, 3 million advertisement impressions resulted in 4 enrolled participants, resulting in an extremely low yield [28]. The yield in the current study, namely 5.1% (287/5644) of the MyChart invitations, was substantially higher than the yields from mass mailing and social media advertising used in our prior study targeting cancer survivors [28]. The success of EHR-based recruitment is aligned with supporting physicians who have an ongoing need for multicomponent weight loss programs to which they can refer patients [29]. As such, the ASPIRE programs hold the potential to support health behavior change in the context of a health care system.

The recruitment approach had some limitations. Although we recruited across the 5 geographic regions in Maryland, we primarily used a single EHR system for recruitment creating a potential for selection bias. For example, the prevalence of smoking in the current sample (4.1%) is lower than that in Maryland (10.1%) and for cancer survivors (12.2%) [30,31]. Alternative recruitment approaches, such as provider referrals may be needed to better engage smokers and thus support this high-risk group. Although the EHR recruitment provided a strong yield in this study, relying on EHR from a single organization would not reach all cancer survivors in Maryland. A comprehensive approach to supporting cancer survivors in Maryland would require multiple recruitment channels. This study’s materials were developed using a theoretical framework, however, those in the ASWL program may have relied on the app rather than this study’s materials for guidance. A review of the theory-based content of weight loss apps indicated the theory-based for Lose it! was low. However, compared to other reviewed apps, Lose it! had the most robust theoretical background and incorporated an essential theory-based element of self-tracking [32].

Our study design also had several strengths. The weight loss programs were adapted from programs previously demonstrated as effective in the general population and among cancer survivors [12-14]. The all-remote design ensured study continuation during COVID-19 restrictions and is aligned with the concept of large-scale dissemination. The ASWL and SDWL programs are readily scalable. Unlike a clinical trial where only those who qualify for the trial receive support, the pragmatic approach in the current study offered weight loss options for individuals with varying interests. Had this study only implemented intensive interventions, for example, the CSWL program, as accomplished in our prior trials, the 233 individuals in the SDWL and ASWL programs would not have received weight loss support. By providing a range of programs and working with participants to find an approach they preferred, the current pragmatic design doubled the reach compared to a more traditional randomized control trial. This collection of programs has the potential to be offered on a larger scale and serve cancer survivors throughout Maryland. In conclusion, these results support EHR-based recruitment efforts as a pragmatic approach to reach and enroll cancer survivors into remotely delivered, weight loss programs.

## Abbreviations

ASWL: app-supported weight loss
CSWL: coach-supported weight loss
EHR: electronic health record
REDCap: Research Electronic Data Capture
SDWL: self-directed weight loss

## Data Availability

The data sets generated or analyzed during this project are available from the corresponding author upon reasonable request.
