# Correction: Behavioral Weight Loss Programs for Cancer Survivors Throughout Maryland: Protocol for a Pragmatic Trial and Participant Characteristics

**DOI:** 10.2196/63678

**Published:** 2024-10-07

**Authors:** Gerald J Jerome, Lawrence J Appel, Linda Bunyard, Arlene T Dalcin, Nowella Durkin, Jeanne B Charleston, Norma F Kanarek, Michael A Carducci, Nae-Yuh Wang, Hsin-Chieh Yeh

**Affiliations:** 1 Department of Kinesiology Towson University Towson, MD United States; 2 Department of Medicine Johns Hopkins University Baltimore, MD United States; 3 Department of Epidemiology Johns Hopkins University Baltimore, MD United States; 4 Department of Environmental Health and Engineering Johns Hopkins University Baltimore, MD United States; 5 Sidney Kimmel Comprehensive Cancer Center Johns Hopkins University Baltimore, MD United States; 6 Department of Biostatistics Johns Hopkins University Baltimore, MD United States

In “Behavioral Weight Loss Programs for Cancer Survivors Throughout Maryland: Protocol for a Pragmatic Trial and Participant Characteristics” (JMIR Res Protoc. 2024 Jun 12:13:e54126), the authors noted one error.

In the originally published article, Figure 1 included additional information regarding the follow-up time points rather than focussing on baseline data. The originally published figure is available in Multimedia Appendix 1. Figure 1 has been corrected as follows:

**Figure 1:**
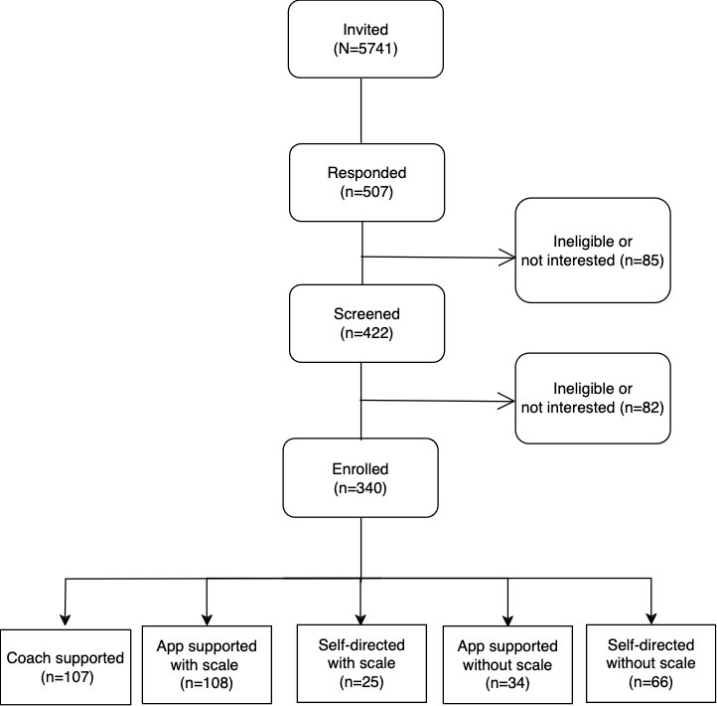
Flow of study participants.

The correction will appear in the online version of the paper on the JMIR Publications website on October 7, 2024 together with the publication of this correction notice. Because this was made after submission to PubMed, PubMed Central, and other full-text repositories, the corrected article has also been resubmitted to those repositories.

## Appendix

Multimedia Appendix 1 Original version of Figure 1.

