# Supplementary figures and images for: Correction: Behavioral Weight Loss Programs for Cancer Survivors Throughout Maryland: Protocol for a Pragmatic Trial and Participant Characteristics
Source: JMIR Res Protoc. 2024 Oct 7;13:e63678. doi: 10.2196/63678 (PMC11514525; doi:10.2196/63678)

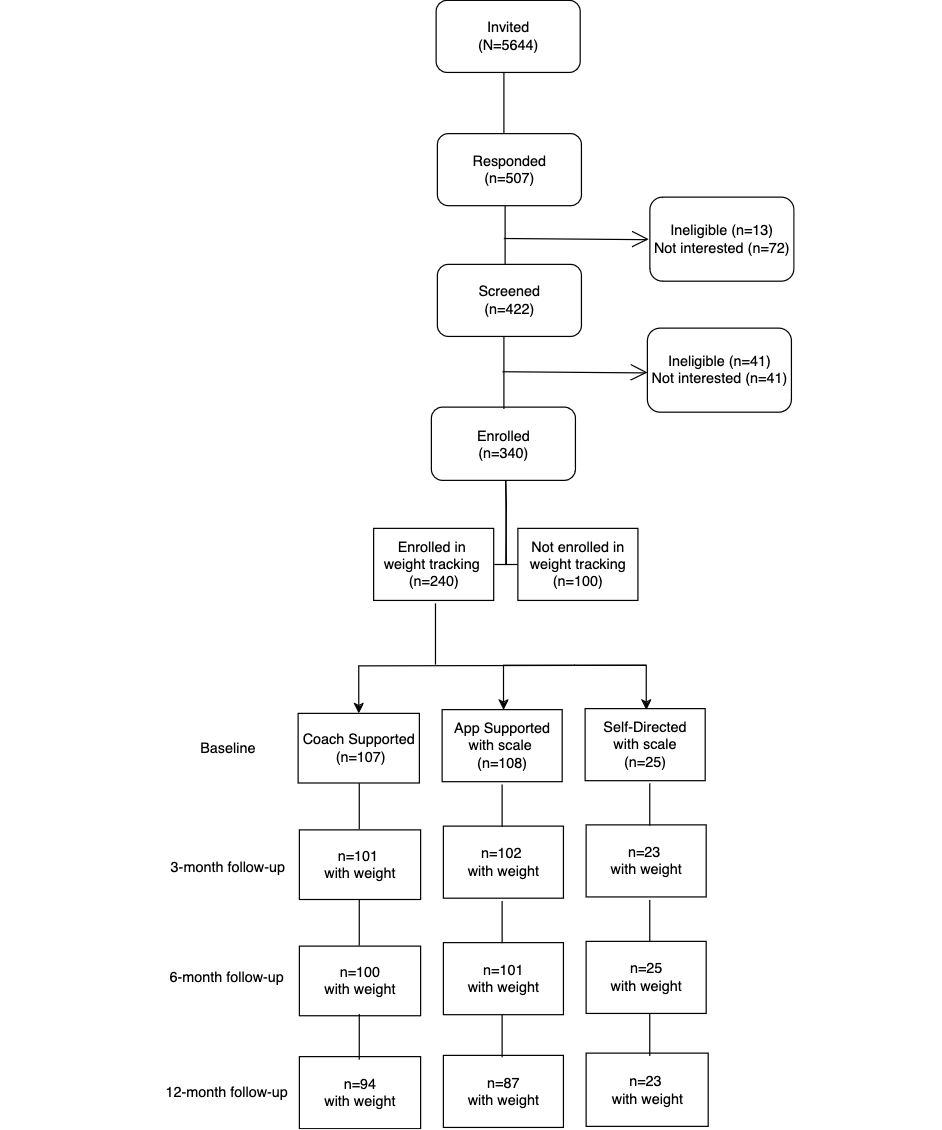

Supplement: Multimedia Appendix 1 [file resprot_v13i1e63678_app1.png]
